# Application of targeted metagenomic next-generation sequencing in pneumonia patients

**DOI:** 10.1128/spectrum.01713-24

**Published:** 2025-06-23

**Authors:** Guanjun Ren, Liyun Ma, Chunliang Yan, Qishan Xue, Huijuan Zhang, Wei Wang, Xiyan Ren, Yun Lei, Shaofei Li, Yafeng Liu, Qingyue Zheng, Shigang Wei, Yue Zhang, Xiao Wang

**Affiliations:** 1 Pulmonary and Critical Care Medicine, Beijing Aerospace General Hospital 605506, Beijing, China; City of Hope Department of Pathology, Duarte, California, USA

**Keywords:** targeted metagenomic next-generation sequencing, capture hybridization, multiplex PCR, pneumonia, mixed infections

## Abstract

After the coronavirus disease 2019 (COVID-19) pandemic, the incidence rate of mixed infections, especially in critically ill patients with severe pneumonia, increases due to the immunity gap and has also been proven to be associated with mortality, increasing the difficulty in accurate and rapid diagnoses. Here, we evaluated the performance of targeted metagenomic next-generation sequencing (mNGS), including capture hybridization-based mNGS (chNGS) and multiplex PCR-based targeted mNGS (tNGS), in diagnosing pneumonia. Patients admitted to the Pulmonary and Critical Care Medicine, Beijing Aerospace General Hospital, and diagnosed as suspected pulmonary infections from April 2022 to March 2024 were retrospectively evaluated, and 110 patients were finally enrolled. According to the final comprehensive clinical diagnoses, there were 99 patients diagnosed with definite infectious diseases. Single infections accounted for 58.6% of these patients (58/99), while mixed infections occurred in about half of these patients (41.4%, 41/99) and were found in most of the death cases. *Pseudomonas aeruginosa* (*n* = 22), *Pneumocystis jirovecii* (*n* = 15), and severe acute respiratory syndrome coronavirus 2 (*n* = 13) were found to be the most common bacterial, fungal, and viral pathogens, respectively. Taking final comprehensive clinical diagnoses as the reference standard, the total coincidence rate (TCR) of chNGS can reach up to 64.1% (95% confidence interval [CI], 54.8%–73.3%), while the TCR of conventional methods was only 39.8% (95% CI, 30.4%–49.3%). The performance of tNGS was slightly superior to that of chNGS, while chNGS yielded more false-negative results, especially for viral detection. Additionally, chNGS combined with tNGS can improve the TCR to 81.3% (95% CI, 62.1%–100.0%).

**IMPORTANCE:**

This is the first report on evaluating the performance of capture hybridization-based metagenomicnext-generation sequencing (chNGS), multiplex PCR-based targeted mNGS (tNGS), and conventional methods in diagnosing pneumonia. Our findings emphasized the importance of chNGS and tNGS in diagnosing, managing, and ruling out infections, and an era of widespread application of regional tNGS in monitoring and diagnosing infections with high sensitivity and low economic burden on patients can be expected.

## INTRODUCTION

With the development in the treatment, morbidity and mortality rates of pneumonia decreased worldwide (1–3). However, it was reported that pneumonia caused about 2.5 million deaths in 2019, and it is still the fourth highest global cause of death (4). Additionally, pneumonia also imposes a substantial economic burden, especially for the patients who require hospitalization or have other underlying diseases (5, 6). This economic burden significantly increases the mortality rate of pneumonia in low-income countries, while the lowest rates of mortality and morbidity are found in developed countries (6, 7). Empirical therapy is often ineffective in treating patients with atypical pneumonia (8). Most importantly, after the pandemic of the coronavirus disease 2019 (COVID-19), the incidence rate of mixed infections, which was associated with mortality (9, 10), inevitably increases (11–13). Therefore, rapid and accurate identification of pathogens is very important for reducing unnecessary treatment, economic burden, and the rates of morbidity and mortality in both low-income and developed countries.

Long turnaround time, limited detection range, low positive rate, and low specificity significantly limited the widespread application of conventional methods (CTMs), including microbial culture (14), microscopic smear (15), polymerase chain reaction (PCR) (16), histopathology (17), and serological antibody detection (18), in rapid and accurate identification of pathogens. Unbiased metagenomic next-generation sequencing (mNGS) has been widely used to diagnose various infections, such as bloodstream infection, abdominal cavity infection, and central nervous system infection (17, 19, 20), and has become a promising detection method for infectious diseases (21).

The sensitivity of mNGS positively correlated with the ratio of pathogen nucleic acid to human nucleic acid (22), and the biggest challenge for the accurate detection of mNGS in limited sequencing depth is how to reduce the high ratio of human nucleic acid. During the mNGS pipeline, the most commonly used host-depleted enrichment (23, 24), such as the differential lysis method, can filter human nucleic acid (23), increasing the ratio of pathogen nucleic acid (23–25) at the expense of some viruses, parasites, and bacteria (23). Apart from host-depleted enrichment, some mNGS methods using targeted enrichments have been developed, such as capture hybridization-based mNGS (chNGS) (26) and multiplex PCR-based targeted mNGS (tNGS) (27, 28). However, there were few studies on evaluating the performance in diagnosing pneumonia between chNGS and tNGS.

Therefore, our study retrospectively enrolled patients with suspected pulmonary infections, evaluated the diagnostic value of chNGS in the diagnosis of pulmonary infections by comparing the diagnostic performance between chNGS and CTM against the final comprehensive clinical diagnoses, and summarized pathogen profiles in pneumonia patients. Additionally, taking final comprehensive clinical diagnoses as the reference standard, we also summarized the coincidence rates of chNGS and tNGS when testing the same samples.

## MATERIALS AND METHODS

### Patients and sample collection

**Fig 1:**
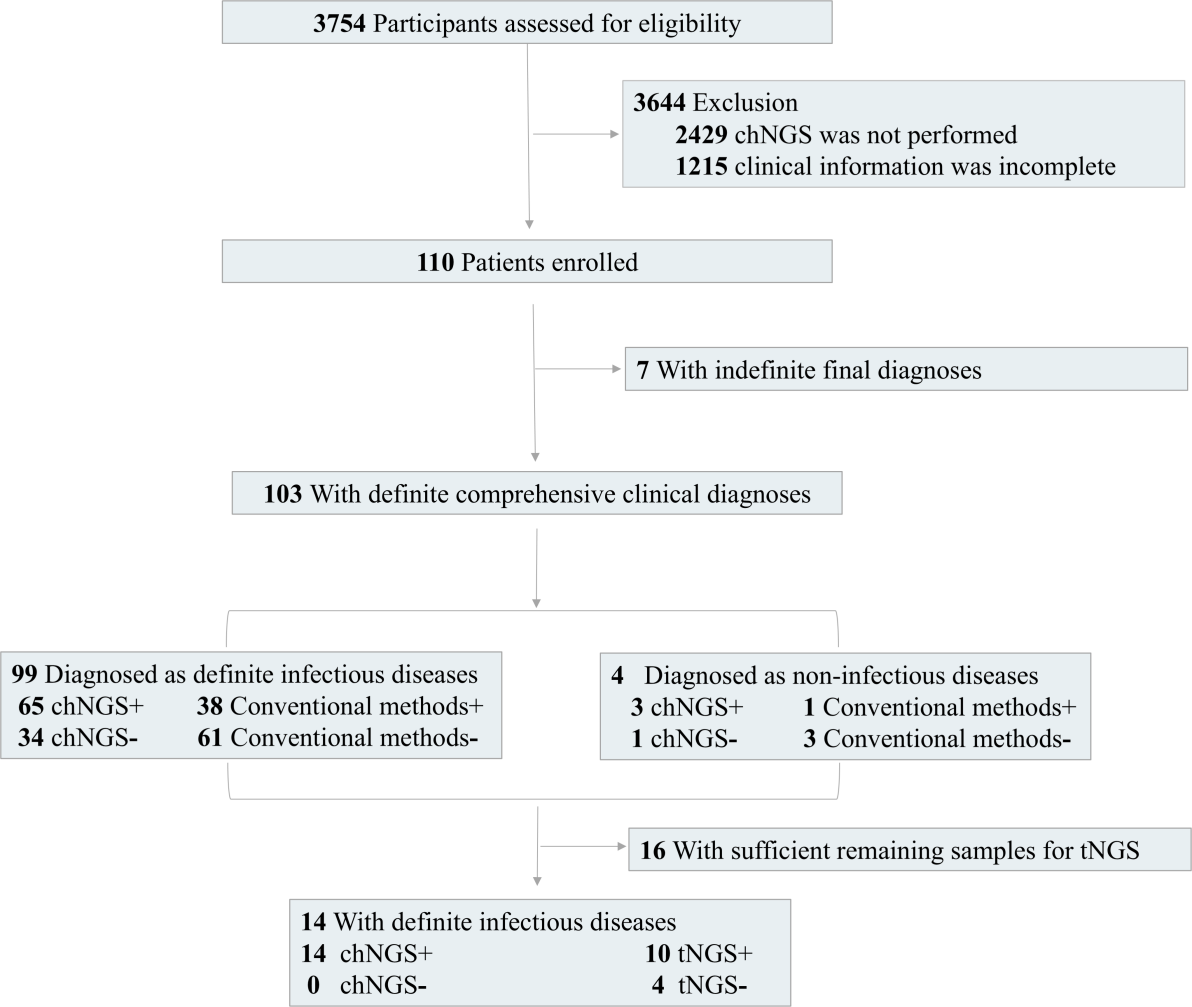
Flow diagram.

A total of 110 patients admitted to Pulmonary and Critical Care Medicine, Beijing Aerospace General Hospital, from April 2022 to March 2024 diagnosed as suspected pulmonary infections were finally enrolled (Fig. 1). All patients with suspected pulmonary infection had abnormal chest imaging results. The inclusion criteria were defined according to the published study (29). Suspected pulmonary infection was considered if the patient had new opacity on imaging examination and at least one of the following symptoms: (i) respiratory distress, (ii) fever, (iii) cough, and (iv) peripheral leukocytosis (>10 × 10^9^/L) or leukopenia (<4 × 10^9^/L). The exclusion criteria were as follows: (i) patients on whom chNGS was not performed and (ii) patients with incomplete clinical and laboratory data. Physical information and clinical characterization were collected. BALF, sputum, or blood samples were collected from patients. Specimens were subjected to CTM and chNGS testing in parallel. CTM, including smear and culture, quantitative PCR, and detection of antibody, was selectively performed according to the severity of patients, and only culture was performed on all enrolled patients. To enhance the reliability of the comparison, only culture results were used for the comparison with chNGS. Additionally, the CURB-65 (confusion, urea, respiratory rate, blood pressure, age ≥65 years) score was used to evaluate the severity of the patient (30).

### chNGS pipeline

Microbial biomass and human cells were separated by centrifuging at 12,000 × *g* for 5 min. We added 1 U Benzonase (Sigma) and 0.5% Tween 20 (Sigma) into the precipitate and then incubated at 37°C for 5 min to eliminate host nucleic acid. Following incubation, 400 µL of terminal buffer was added to stop the reaction. The total nucleic acid was then extracted using a QIAamp UCP Pathogen Mini Kit (Qiagen, Germany). The KAPA low throughput library construction kit (KAPA Biosystems, USA) was used to construct DNA/cDNA library according to the manufacturer’s instructions (31). The constructed library from each sample was used for capture hybridization-based enrichment with microbial probes (SeqCap EZ Library, Roche, USA), followed by sequencing on the Nextseq 550 platform (Illumina, San Diego, USA). On average, about 2 million single-end 50 bp reads were obtained per sample.

### tNGS pipeline

According to the public pathogen databases, the in-house tNGS panel was designed to detect 273 pathogens causing infections in different systems based on multiplex PCR and mNGS. The 273 pathogens included 113 bacteria, 47 fungi, 101 viruses, and 12 parasites. During the sample pretreatment, there was no need to eliminate host nucleic acid. After nucleic acid extraction, multiplex PCR with the designed primers was used to construct libraries. Library concentrations were quantified using Qubit 4.0. Illumina NextSeq platform was used for high-throughput sequencing.

### Bioinformatics analysis

To remove adapters and low-quality, low-complexity, and short reads of <35 bp, raw data generated by the sequencing were filtered (32). To exclude human DNA sequences, reads were mapped to the human reference genome hg38 using bowtie2 (33) to obtain clean reads. And then, the clean reads were blasted against the in-house constructed microbial genome database. To construct the in-house microbial genome database, we downloaded some published databases, including the reference sequence database at the National Center for Biotechnology Information, IMG database, and PATRIC database, and further processed the sequences in the downloaded databases by seeking common ground while reserving differences. The constructed databases contain 25,863 pathogens, including 12,142 bacteria, 2,680 fungi, 10,061 viruses (including DNA and RNA viruses), 654 parasites, 206 mycobacteria, and 120 mycoplasma/chlamydia. Finally, microbial information at the species level can be obtained.

### Interpretation of chNGS/tNGS

As control, negative (“No template” control, NTC) and positive (artificially synthesized nucleic acid standards) controls were also set with the same procedure and bioinformatics analysis. Strictly map reads number (SMRN) and genomic coverage were analyzed. SMRN represents the number of sequences that are strictly aligned with the microorganism at species level. During the interpretation process, positive chNGS/tNGS results were defined as follows:

SMRN of the same microorganism in the sample should be higher than that in the negative control.

1. Bacteria, fungi, mycoplasma, and chlamydia: SMRN  ≥  3.
2. Parasite: SMRN  ≥  100.
3. Viruses, *Mycobacterium*, and *Cryptococcus*: SMRN ≥ 1.

With reference to published study (29), positive chNGS/tNGS results were further confirmed according to whether the pathogens were the most commonly reported pathogens or the infections by the pathogens were in accordance with the clinical features of patients (34, 35).

### Diagnostic assessment

According to laboratory examinations, response of the patients to treatment, chNGS results, and clinical experiences, 2–3 clinical adjudicators (GJR, CLY, and YFL) independently made the final comprehensive clinical diagnoses and defined the causative pathogens. Subsequently, taking final comprehensive clinical diagnoses as the reference standard, the enrolled patients were further divided into the indefinite clinical diagnosis group and the definite clinical diagnosis group, including infectious diagnosis and non-infectious diagnosis groups (35), which were used to calculate the sensitivity, specificity, positive predictive value (PPV), negative predictive value (NPV), and total coincidence rate (TCR) of chNGS and tNGS.

### Statistical analysis

Continuous variables were expressed as mean  ±  standard error (SE), and categorical variables were expressed as numbers (percentage). We tested for differences in continuous variables using the t test and categorical variables with the χ test as appropriate. Data analyses were performed using SPSS 22.0 software, and a two-tailed value of *P* of <0.05 was considered to represent a significant difference.

## RESULTS

### Baseline characteristics of enrolled patients

**Fig 2:**
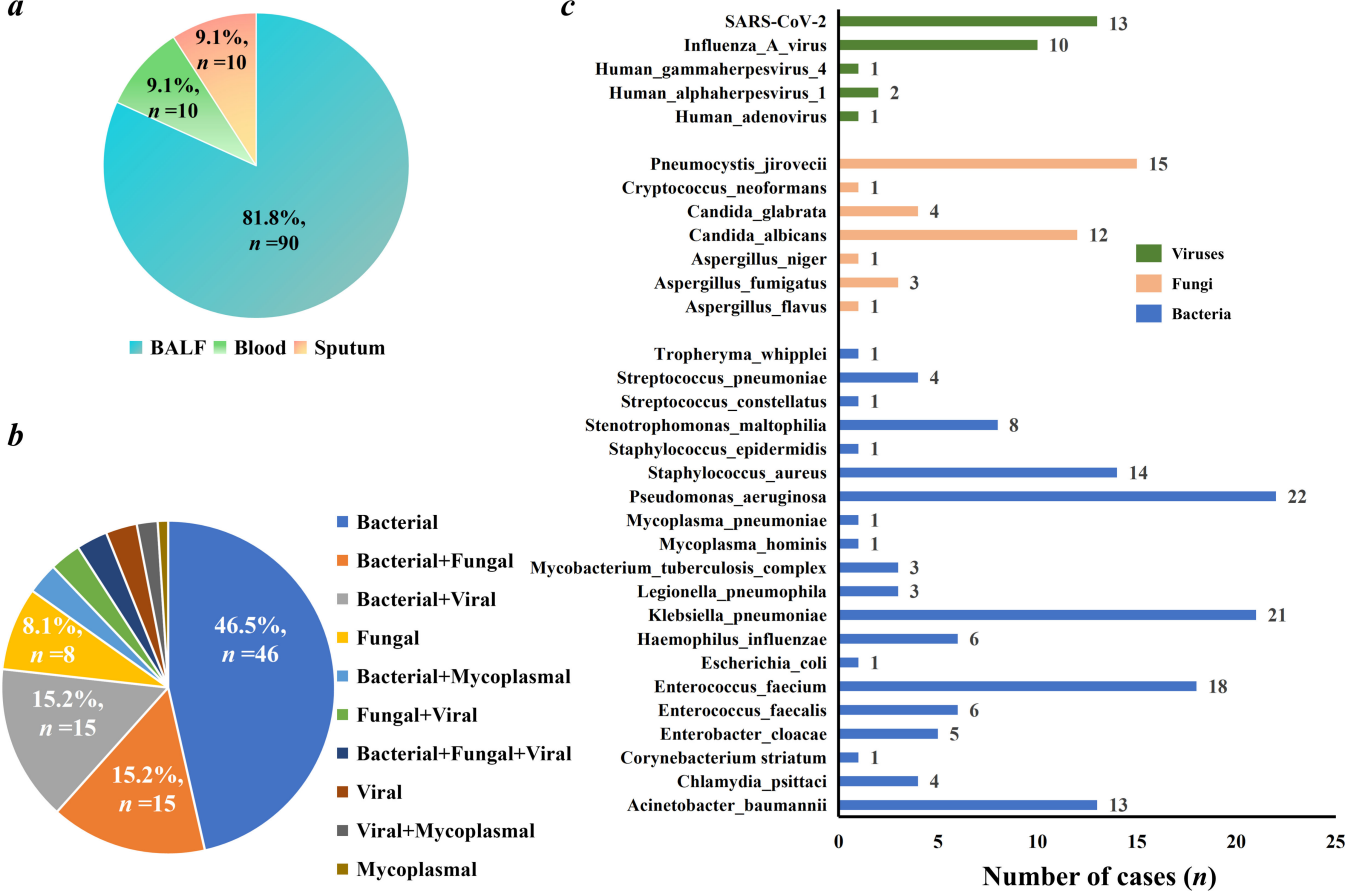
Infection type and pathogen profiles. (**a**) Sample types used for pathogen detection. (**b**) Different types of infections found in pneumonia. (**c**) Pathogen profiles. The number on the right side of the pathogen bar is the number of patients with the detection of the pathogen. SARS-CoV-2 represents severe acute respiratory syndrome coronavirus 2.

In this study, we finally enrolled a total of 110 patients (87 males [79.1%) and 23 females (20.9%)) with suspected pulmonary infection (Table 1). Most of the patients were over 40 years old (95.4%), while more than half of the patients was >70 years old (*n* = 60, 54.5%). The 57.3% of enrolled patients (*n* = 63) had a CURB-65 score of 0–1, while the CURB-65 score of 29 patients and 18 patients was 2 and ≥3, respectively. Among them, 85.5% of enrolled patients (*n* = 94) had underlying diseases, including hypertension (*n* = 35, 31.8%), cerebral infarction (*n* = 14, 12.7%), heart disease (*n* = 13, 11.8%), cancer (*n* = 10, 9.1%), diabetes (*n* = 8, 7.3%), chronic obstructive pulmonary disease (*n* = 4, 3.6%), gallstones (*n* = 2, 1.8%), cerebral hemorrhage (*n* = 2, 1.8%), hepatitis B (*n* = 1, 0.9%), systemic lupus erythematosus (*n* = 1, 0.9%), allergic rhinitis (*n* = 1, 0.9%), pulmonary interstitial lesions (*n* = 1, 0.9%), myocardial infarction (*n* = 1, 0.9%), and asthma (*n* = 1, 0.9%). For further diagnoses, samples were collected from 110 patients for chNGS and CTM testing, including 90 BALF, 10 blood, and 10 sputum samples (Fig. 2a). According to final comprehensive clinical diagnoses, we divided enrolled patients into definite (*n* = 103) and indefinite (*n* = 7) clinical diagnosis groups (Fig. 1).

**TABLE 1:** Baseline characteristics of enrolled patients

Characteristic	No. of cases	Percentage (%) of cases
Sex		
Male	87	79.1
Female	23	20.9
Age (years)		
≤40	5	4.5
>40, ≤70	45	40.9
>70	60	54.5
CURB-65 score		
0–1	63	57.3
2	29	26.4
≥3	18	16.4
Underlying illness		
Hypertension	35	31.8
Cerebral infarction	14	12.7
Heart disease	13	11.8
Cancer	10	9.1
Diabetes	8	7.3
Chronic obstructive pulmonary disease	4	3.6
Gallstones	2	1.8
Cerebral hemorrhage	2	1.8
Hepatitis B	1	0.9
Systemic lupus erythematosus	1	0.9
Allergic rhinitis	1	0.9
Pulmonary interstitial lesions	1	0.9
Myocardial infarction	1	0.9
Asthma	1	0.9

### Infection type and pathogen profiles

According to final comprehensive clinical diagnoses, there were 99 patients diagnosed as definite infectious diseases and four patients diagnosed as non-infectious diseases (Fig. 1). Among the patients with pulmonary infections, single infection accounted for 58.6% of these patients (58/99), and mixed infections occurred in about half of these patients (41.4%, 41/99) (Fig. 2b). For single infection, bacterial infection (*n* = 46, 46.5%) was found to be the most common infection type, followed by fungal infection (*n* = 8, 8.1%), viral infection (*n* = 3, 3.0%), and mycoplasmal infection (*n* = 1, 1.0%), while more than two kinds of bacterial pathogens were diagnosed as causative pathogens in more than half of patients with bacterial infection (25/46). Additionally, mixed infections included bacterial-fungal co-infection (*n* = 15, 15.2%), bacterial-viral co-infection (*n* = 15, 15.2%), bacterial-mycoplasmal co-infection (*n* = 3, 3.0%), fungal-viral co-infection (*n* = 3, 3.0%), bacterial-fungal-viral co-infection (*n* = 3, 3.0%), and viral-mycoplasmal co-infection (*n* = 2, 2.0%). The above results show that after the COVID-19 pandemic, there was an increased incidence rate of mixed infections in pneumonia patients.

The pathogen profiles were also summarized (Fig. 2c). For bacterial pathogens, *Pseudomonas aeruginosa* was diagnosed as the causative pathogen in 22 patients, followed by *Klebsiella pneumoniae* (*n* = 21), *Enterococcus faecium* (*n* = 18), *Staphylococcus aureus* (*n* = 14), *Acinetobacter baumannii* (*n* = 13), and *Stenotrophomonas maltophilia* (*n* = 8). *Pneumocystis jirovecii* (*n* = 15) was found to be the most common fungal pathogen, and there were 12 and five patients respectively infected by *Candida albicans* and *Aspergillus*. Additionally, viral infection was mainly caused by severe acute respiratory syndrome coronavirus 2 (SARS-CoV-2) (*n* = 13) and Influenza A virus (*n* = 10).

### Diagnostic value of chNGS and CTM

**Fig 3:**
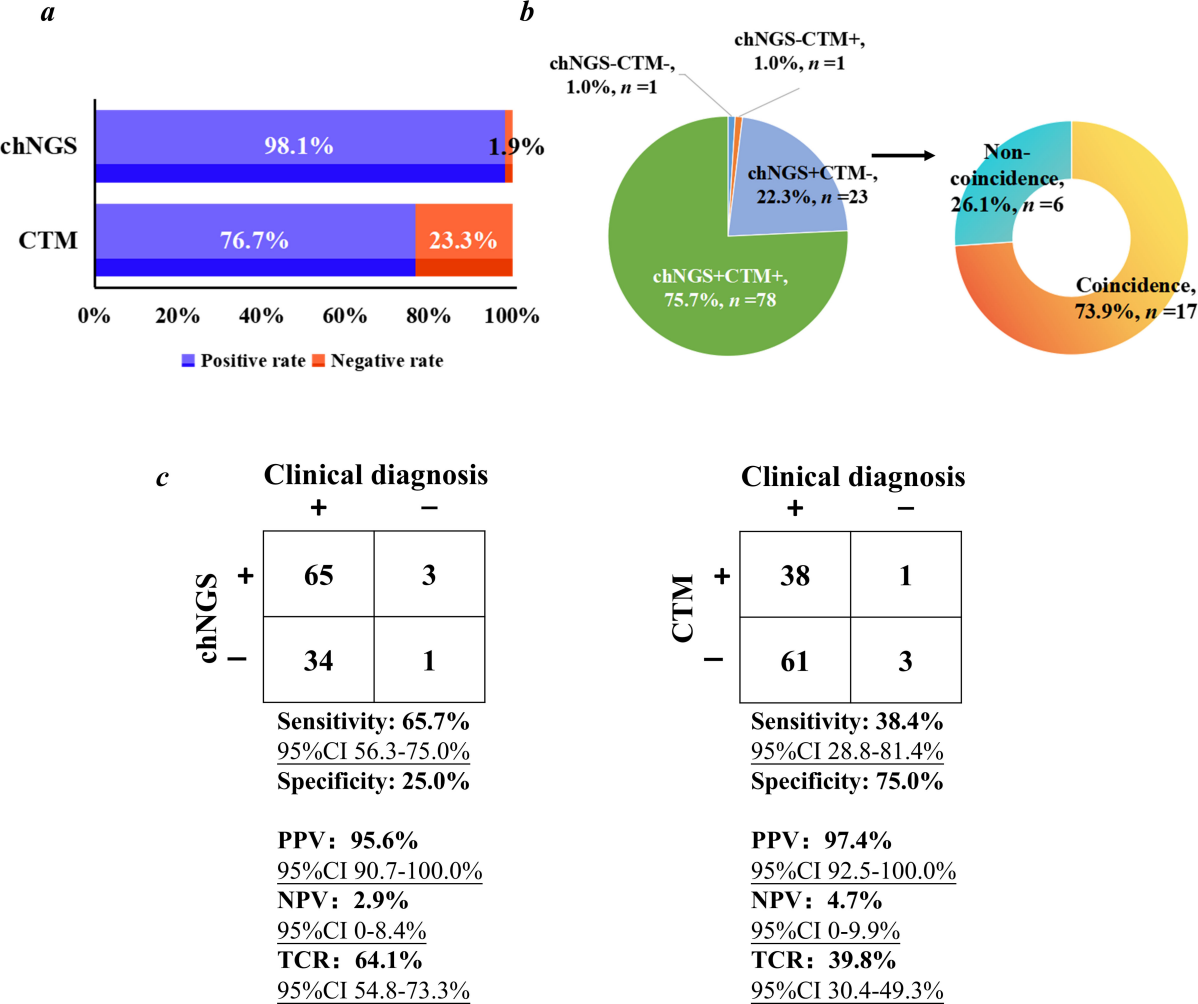
Performance comparison between chNGS and CTM. (**a**) Positive rates of chNGS and CTM. (**b**) Summaries of the number of cases with positive or negative results revealed by chNGS and CTM. “+” and “−” represent positive and negative results, respectively. (**c**) Performance of chNGS and CTM. PPV, NPV, and TCR represent positive predictive value, negative predictive value, and total coincidence rate, respectively.

In our study, both chNGS and CTM were simultaneously performed on the whole of enrolled patients. We calculated the positive rates of chNGS and CTM in 103 patients with definite clinical diagnoses (Fig. 3a). It was found that chNGS can detect microbes from most of the patients (98.1%, 101/103), while the positive rate of CTM was 76.7% (79/103). There were 78 patients (75.7%) with both chNGS and CTM positive, and 23 patients (22.3%) with chNGS positive and CTM negative, while for the two patients with chNGS negative, CTM can only detect microbes from one patient (Fig. 3b). Additionally, for the patients with chNGS positive and CTM negative, more than 73% of those patients (17 out of 23) can be directly diagnosed only by chNGS results.

Taking final comprehensive clinical diagnoses as the reference standard, the performance of chNGS and CTM in diagnosing infections was further calculated (Fig. 3c). We found that the sensitivity of chNGS (65.7%; 95% confidence interval [CI], 56.3%–75.0%) was significantly higher than that of CTM (38.4%; 95% CI, 28.8%–81.4%), while the specificity showed the opposite trend. Both chNGS (90.9%; 95.6% CI, 90.7%–100.0%) and CTM (97.4%; 95% CI, 92.5%–100.0%) had high PPV, while the NPV was low (chNGS, 2.9% [95% CI, 0%–8.4%] and CTM, 4.7% [95% CI, 0%–9.9%]). In addition, more than 64% of chNGS results (TCR, 64.1% [95% CI, 54.8%–73.3%]) were consistent with the final clinical diagnoses, while the TCR of CTM was only 39.8% (95% CI, 30.4%–49.3%). The above results unravel that chNGS was proven to be more effective than CTM in diagnosing mixed infections among pneumonia patients.

### Performance comparison between chNGS and tNGS

Given the economic burden on patients and the high sensitivity of tNGS, we further performed tNGS on the 16 patients with sufficient remaining samples after conducting chNGS and CTM tests to evaluate the performance of chNGS and tNGS. It was found that tNGS and chNGS have the same sensitivity (Fig. S1). For the two patients with non-infectious diseases, chNGS produced false-positive results, while tNGS did not detect any pathogens. TCR of tNGS (68.8% [95% CI, 46.0%–91.5%]) was slightly higher than that of chNGS (56.3% [95% CI, 31.9%–80.6%]). Additionally, chNGS combined with tNGS can improve the sensitivity and TCR to 78.6% (95% CI, 57.1%–100.0%) and 81.3% (95% CI, 62.1%–100.0%). The above results indicate that tNGS could be considered as a supplement to mNGS or chNGS in diagnosing mixed pulmonary infections.

**Fig 4:**
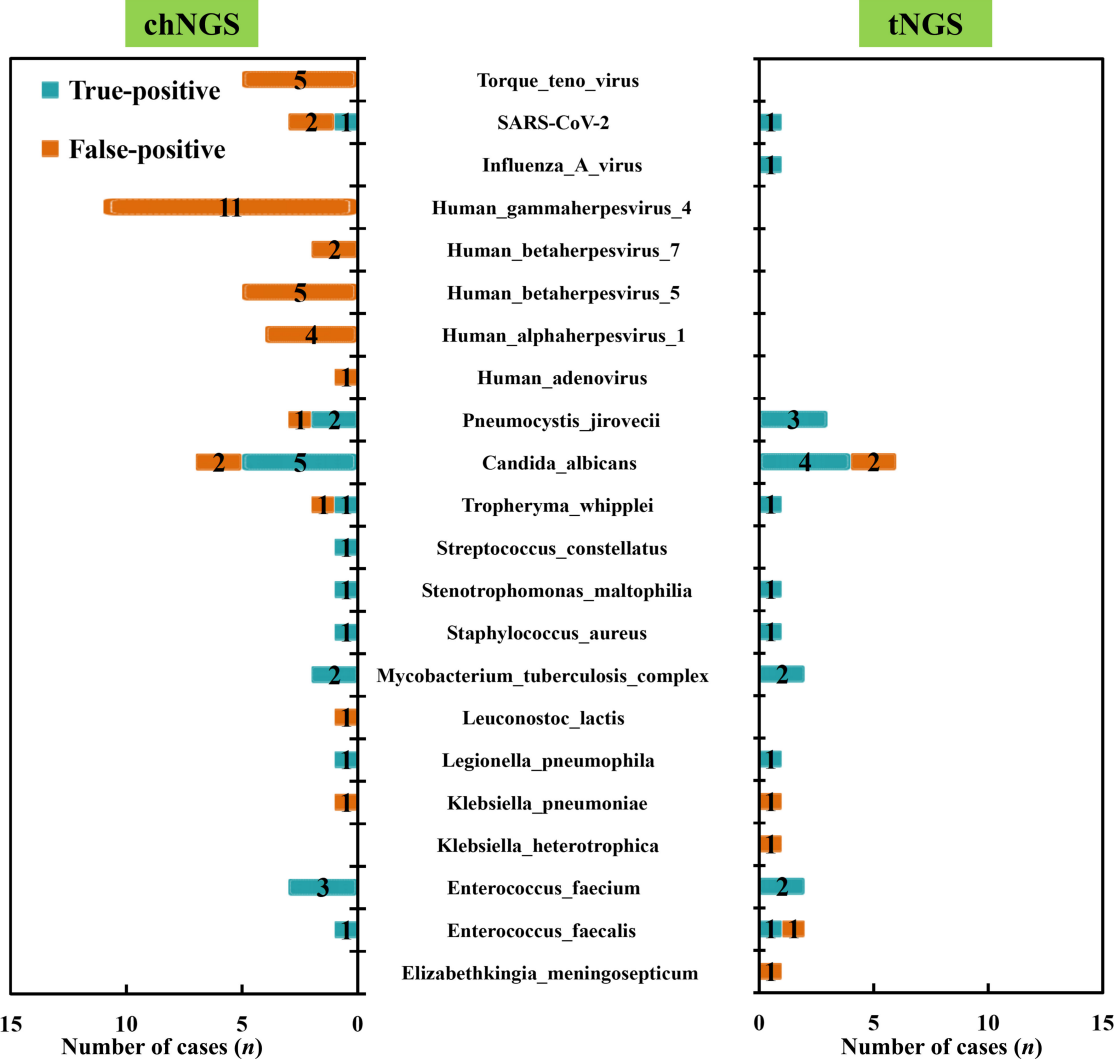
Diagnostic value of chNGS and tNGS from the perspective of pathogens. The number on the pathogen bar is the number of the patients with true-positive or false-positive results.

From the perspective of causative pathogens, we summarized the true-positive and false-positive results of both chNGS and tNGS against final comprehensive clinical diagnoses to further compare the diagnostic value between chNGS and tNGS (Fig. 4; Table S1). If the detected microbe by chNGS or tNGS was the causative pathogen, we considered it a true-positive result, while the other microbes were classified into false-positive results. The most viruses detected by chNGS, including human gammaherpesvirus 4 (*n* = 11), human betaherpesvirus 5 (*n* = 5), Torque teno virus (*n* = 5), and human alphaherpesvirus 1 (*n* = 4), were proven to be false-positive results, while those viruses were not detected by tNGS at all. Influenza A virus infection of one patient was only found by tNGS, rather than chNGS. chNGS and tNGS showed similar performance in *C. albicans* and *P. jirovecii* detection. The most bacterial pathogens detected by both chNGS and tNGS were defined as causative pathogens. However, *Streptococcus constellatus* infection (*n* = 1) was only found by chNGS, while *Klebsiella heterotrophica* (*n* = 1) and *Elizabethkingia meningosepticum* (*n* = 1), only detected by tNGS, were proven to be false-positive results.

### Infection type and final outcome in patients with different CURB-65 scores

**Fig 5:**
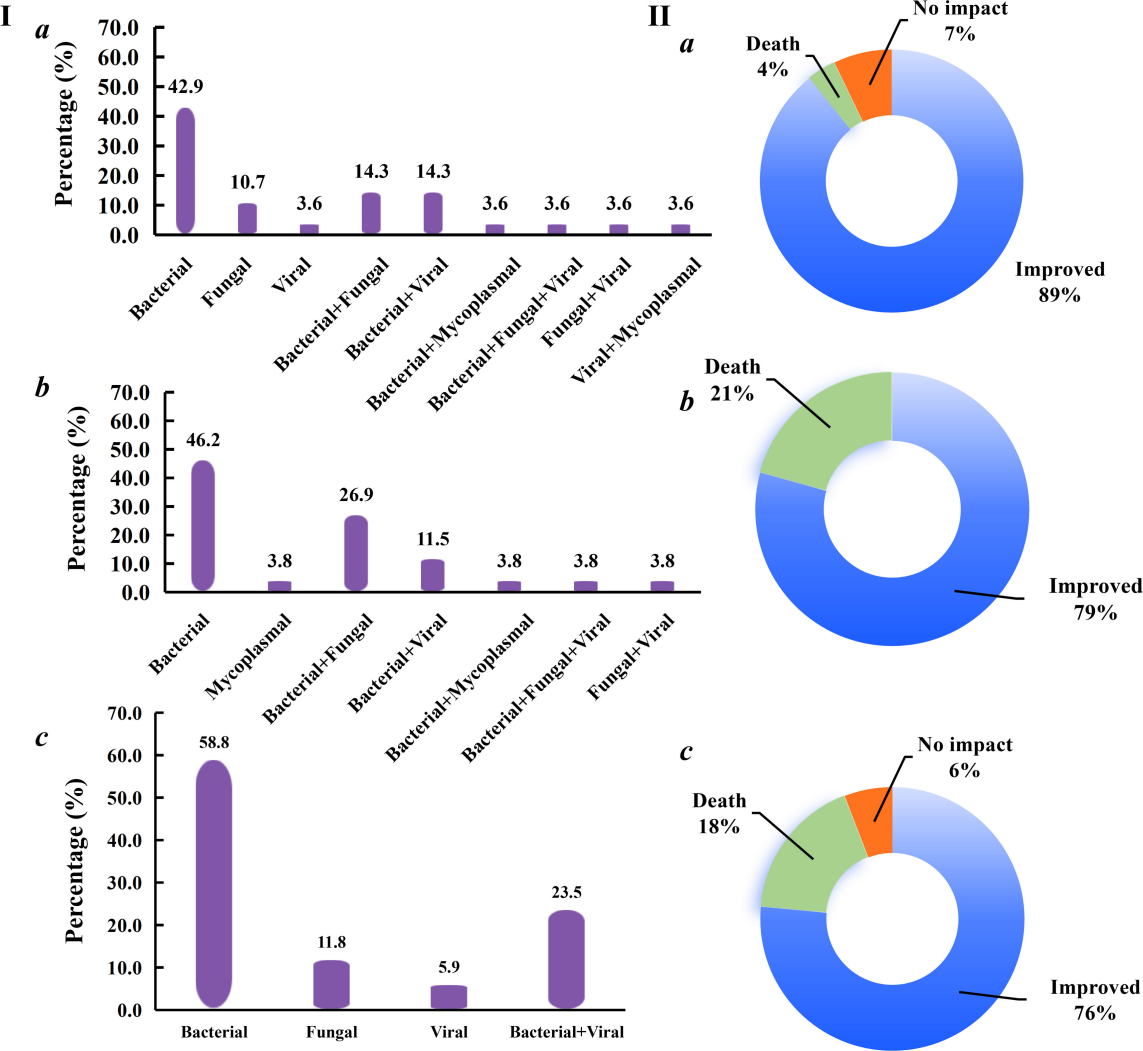
Mixed infections and final outcomes of patients with different CURB-65 scores. Based on the CURB-65 scores, we divided the enrolled patients into mild (CURB-65 scores of 0–1), moderate (CURB-65 score of 2), and severe (CURB-65 scores of ≥3) groups. (I) Infection types in mild (**a**), moderate (**b**), and severe (**c**) groups. (II) Final outcomes in mild (**a**), moderate (**b**), and severe (**c**) groups.

Based on the CURB-65 scores, we divided the enrolled patients into mild (CURB-65 scores of 0–1), moderate (CURB-65 score of 2), and severe (CURB-65 scores of ≥3) groups. Most of the patients in the three groups were mainly caused by single infection, and bacterial infection was found to be the most common infection type (42.9%, 46.2%, and 58.8% in mild [Fig. 5Ia], moderate [Fig. 5Ib], and severe [Fig. 5Ic] groups, respectively). Additionally, more than 76% of patients were improved in the three groups with reference to chNGS results (Fig. 5II). The highest mortality rate was found in the moderate group (20.7%, Fig. 5IIb), followed by the severe group (17.6%, Fig. 5IIc) and the mild group (3.6%, Fig. 5IIa). Most importantly, among the six patients with death outcome in the moderate group, five cases were diagnosed as mixed infections, and the two deaths in the mild group were also found to be mixed infections, while only bacterial infection was found in the three deaths of the severe group (Table S2). The above results further emphasized the important role of mixed infections in influencing the final outcome of pneumonia patients.

## DISCUSSION

To the best of our knowledge, this is the first report on evaluating the performance of chNGS, tNGS, and CTM in diagnosing pneumonia. We found that mixed infections occurred in 41.4% of enrolled patients. Bacterial and fungal infections were mainly caused by *P. aeruginosa* and *P. jirovecii*, respectively. Additionally, chNGS was proven to be more effective than CTM in diagnosing mixed infections among pneumonia patients. The performance of tNGS was slightly superior to that of chNGS. Compared with chNGS, tNGS might be more suitable for ruling out viral pneumonia. It is crucial for us to focus on high-incidence mixed infections in order to improve patient outcomes. In addition, given the low detection rates of CTM and the difficulty in cultivating fungi and viruses, we proposed that chNGS or tNGS should be considered as preferred detection in risk pneumonia patients.

In our study, tNGS demonstrated the potential to perform similarly to chNGS in patients with pneumonia. More and more studies (35–38) have proven that compared with mNGS, low detection efficiencies and accuracies of CTM, such as culture (39), hindered comprehensive detection of pathogens. Under the limited sequencing depth, improving the ratio of pathogen DNA to human DNA can let more microbial sequences be detected, increasing the sensitivity of mNGS (22, 40). Although capture probe enrichment for pathogens has been found to decrease the fraction of sequence classified as human (40, 41), the ratio of human nucleic acid in the generated data of chNGS was still more than 90% (Fig. S2). However, without a host-depleted process (23, 24), constructing a library by multiplex PCR with primers designed to specifically target desired pathogens can only yield microbial products using total nucleic acid extracted, and only microbial sequences were generated by tNGS (42). Additionally, host-depleted processes may incorrectly result in the loss of some viruses, parasites, and bacteria (25, 43), such as hindering the detection of *P. aeruginosa* (24) and *Mycobacterium tuberculosis* (23). Accordingly, the differences in the wet lab pipeline between tNGS and chNGS also led to the differences in their performance.

Currently, tNGS with high sensitivity has reduced the economic burden on patients, and its extensive application can be expected. However, some of the most common pathogens were not detected or partially detected in some cases using the tNGS pipeline in our study (Table S1), taking final comprehensive clinical diagnoses as the reference standard. Given the high sensitivity of PCR and the high throughput of mNGS, pathogens with predesigned primers in the panel (44, 45) could be definitely detected by tNGS. Actually, when more primers targeting a broader range of pathogens are added to the panels, it results in the production of more primer dimer species, thereby reducing the mapping rate of tNGS (46) and increasing the likelihood of missing the detection of certain pathogens. Additionally, given the epidemiology of pathogens characterized by geographical specificity (47), rarity (48), and novelty (49, 50), we proposed that designing and developing regional tNGS can be performed (46, 51), and an era of widespread application of regional tNGS in diagnosing and monitoring infections with high sensitivity and low economic burden on patients can be expected.

Given the differences in methods, we should choose mNGS, chNGS, or tNGS based on the specific situation and research objectives. For pathogen detection in patients with severe infections or non-specific infections, mNGS is more suitable, while for routine infections or general screening, chNGS or tNGS is more appropriate (52). In addition, for the pediatric patients with limited volume samples, the performance of tNGS without host-depleted process (23, 24) must be superior to that of mNGS and chNGS. Due to the targeted enrichment for causative pathogens, the results of chNGS and tNGS lose some microbial information, such as non-pathogenic microorganisms, and mNGS should be applied in microbiome researches, including nasal (53), lung (54), and gut microbiota (55).

We found that mixed infections with a high incidence rate were found to be associated with mortality, which was consistent with the previous studies that mortality of 1918 H1N1 (9) and 2009 H1N1 (10) pandemics was attributed in part to bacterial co-infection. Emerging studies unravel that lung microbiota plays an important role in maintaining lung function and defending against infections (13), and its dynamic balance with low burden can be maintained by mucociliary clearance, cough, as well as innate and adaptive host immune responses (13), while its dysbiosis is associated with clinical consequences as well as development and progression of respiratory diseases. Regular exposure to environmental microbes is crucial for establishing the dynamic microbial-host interface (13). However, strict prevention and control measures from the COVID-19 pandemic caused by the SARS-CoV-2, especially wearing masks, have significantly decreased the diversity of lung microbiota and led humans to lose the opportunity to develop natural immunity against various pathogens (13), resulting in an immunity gap (56). The above evidence provides a good explanation for why the incidence rate of mixed infections in patients with pneumonia, especially for the critically ill patients, inevitably increases (11–13), resulting in poor clinical outcomes.

### Limitations

This study also has some limitations. First of all, a multi-center study should be performed to provide more effective data and avoid intrinsic bias and to comprehensively summarize regional pathogen profiles for further designing and developing of regional tNGS. Second, more cases in which disease was non-infectious are needed to understand the specificity of chNGS and tNGS, particularly ones for non-sterile sources and ones that produce results that may be used for patient care. Third, to further explain why more mixed infections were found during and after the COVID-19 pandemic, metatranscriptomics can be performed to explore the differences in host response and the changes in microbial diversity of lung microbiota before, during, and after the COVID-19 pandemic. Finally, simultaneously applying mNGS, chNGS, and tNGS to samples from a larger cohort of patients could provide more accurate information to guide clinical diagnosis and treatment.

### Conclusions

In our study, we found that the high incidence rate of mixed infections (41.4%) was proven to be associated with mortality. It is crucial for clinicians to focus on mixed infections to improve patient outcomes. *P. aeruginosa*, *P. jirovecii*, and SARS-CoV-2 were found to be the most common bacterial, fungal, and viral pathogens, respectively. The performance of tNGS was slightly superior to that of chNGS, while chNGS yielded more false-negative results, especially for viral detection. Both chNGS and tNGS were proven to be more effective than CTM in diagnosing mixed infections, and tNGS might be more suitable than chNGS for ruling out viral pneumonia. Accordingly, we proposed that widespread application of regional tNGS can be expected.

## Data Availability

Sequencing data were deposited to the National Genomics Data Center under accession numbers CRA016384 and CRA017815. The main data supporting the findings are available within this article. Other data generated and analyzed for this study are available from the corresponding author upon reasonable request.

## SUPPLEMENTAL MATERIAL

Supplemental figures spectrum.01713-24-s0001.docx Figures S1 and S2.

Graphical abstract spectrum.01713-24-s0002.tif Visual diagram of the study.

Table S1 spectrum.01713-24-s0003.xlsx Performance of chNGS and tNGS against the final diagnosis.

Table S2 spectrum.01713-24-s0004.xlsx Infection type, final outcome, and CURB-65 score in patients with definite infectious diseases.

ASM does not own the copyrights to Supplemental Material that may be linked to, or accessed through, an article. The authors have granted ASM a non-exclusive, world-wide license to publish the Supplemental Material files. Please contact the corresponding author directly for reuse.
