# Supplementary material for: Application of targeted metagenomic next-generation sequencing in pneumonia patients
Source: Microbiol Spectr. 2025 Jun 23;13(8):e01713-24. doi: 10.1128/spectrum.01713-24 (PMC12323347; doi:10.1128/spectrum.01713-24)
Supplement: Supplemental figures — Figures S1 and S2. [file spectrum.01713-24-s0001.docx]

**Application of targeted metagenomic next-generation sequencing in** **pneumonia patients**

Guanjun Ren^1^, Liyun Ma^1^, Chunliang Yan*, Qishan Xue, Huijuan Zhang, Wei Wang, Xiyan Ren, Yun Lei, Shaofei Li, Yafeng Liu*, Qingyue Zheng, Shigang Wei, Yue Zhang, Xiao Wang

Pulmonary and Critical Care Medicine, Beijing Aerospace General Hospital, No. 7, Wanyuan North Road, Donggaodi, Fengtai District, Beijing 100076, China.

Guanjun Ren and Liyun Ma are co-first authors of the article.

*Corresponding authors:

Chunliang Yan, Pulmonary and Critical Care Medicine, Beijing Aerospace General Hospital, No. 7, Wanyuan North Road, Donggaodi, Fengtai District, Beijing 100076, China. Email: [yanchunliang2008@126.com](mailto:yanchunliang2008@126.com).

YafengLiu, Pulmonary and Critical Care Medicine, Beijing Aerospace General Hospital, No. 7, Wanyuan North Road, Donggaodi, Fengtai District, Beijing 100076, China. Email: [liuyafeng1995@sina.com](mailto:liuyafeng1995@sina.com).

Running title: tNGS for pneumonia

| 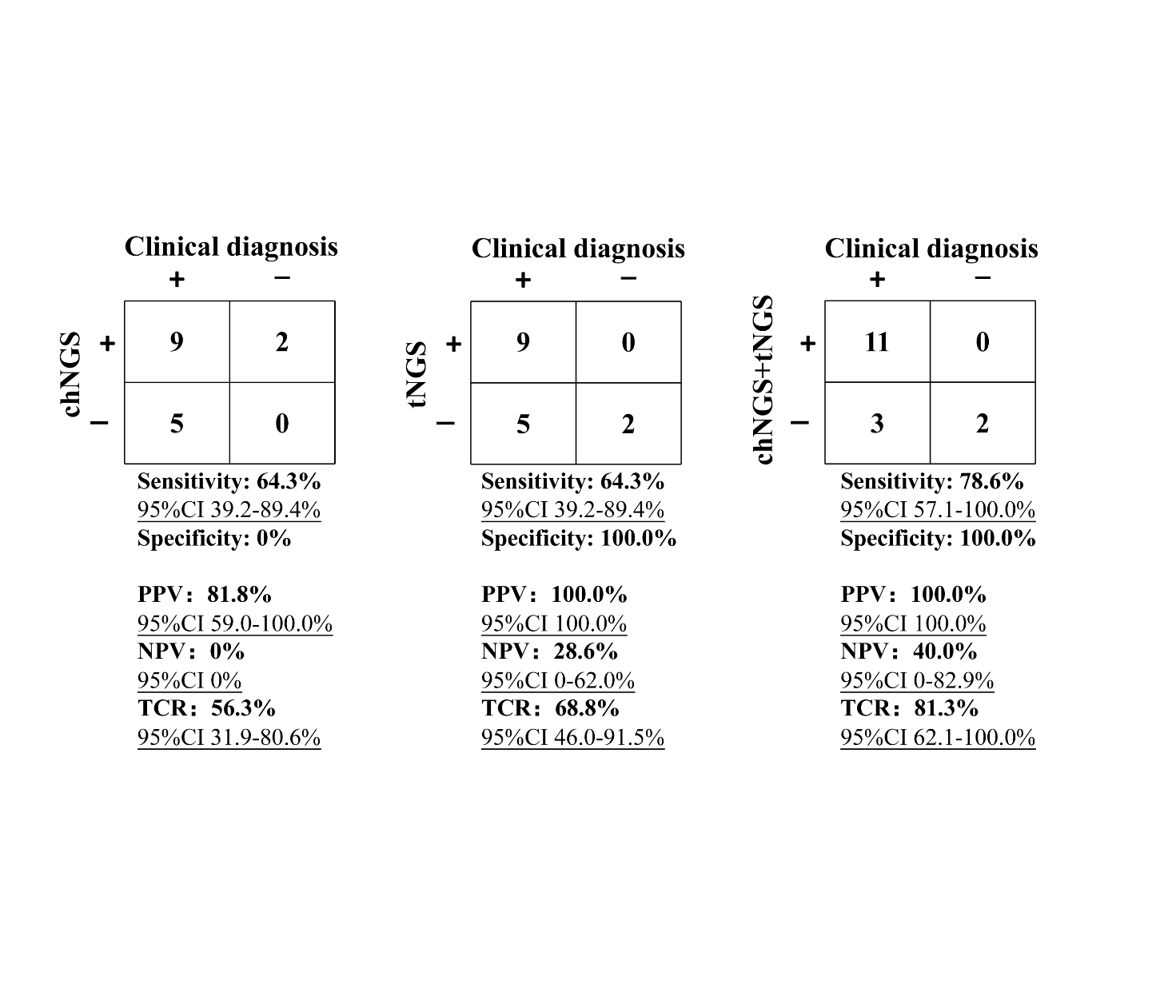 |
| --- |
| Figure S1 Performance of chNGS and tNGS. ‘PPV’, ‘NPV’, and ‘TCR’ represent positive predictive value, negative predictive value, and total coincidence rate, respectively. |

| 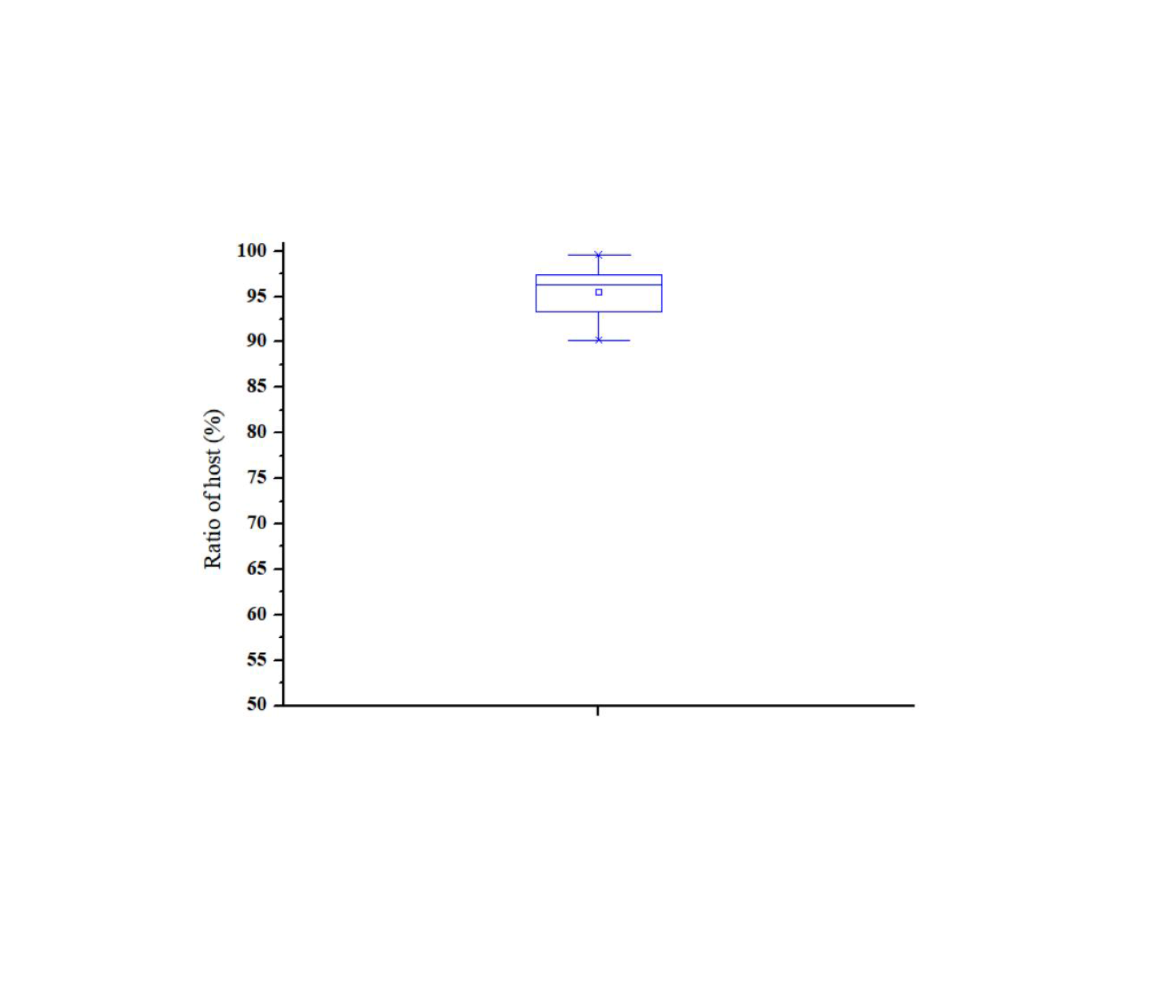 |
| --- |
| Figure S2 The ratio of human nucleic acid in the generated data of chNGS |
