# Supplementary figures and images for: Application of targeted metagenomic next-generation sequencing in pneumonia patients
Source: Microbiol Spectr. 2025 Jun 23;13(8):e01713-24. doi: 10.1128/spectrum.01713-24 (PMC12323347; doi:10.1128/spectrum.01713-24)

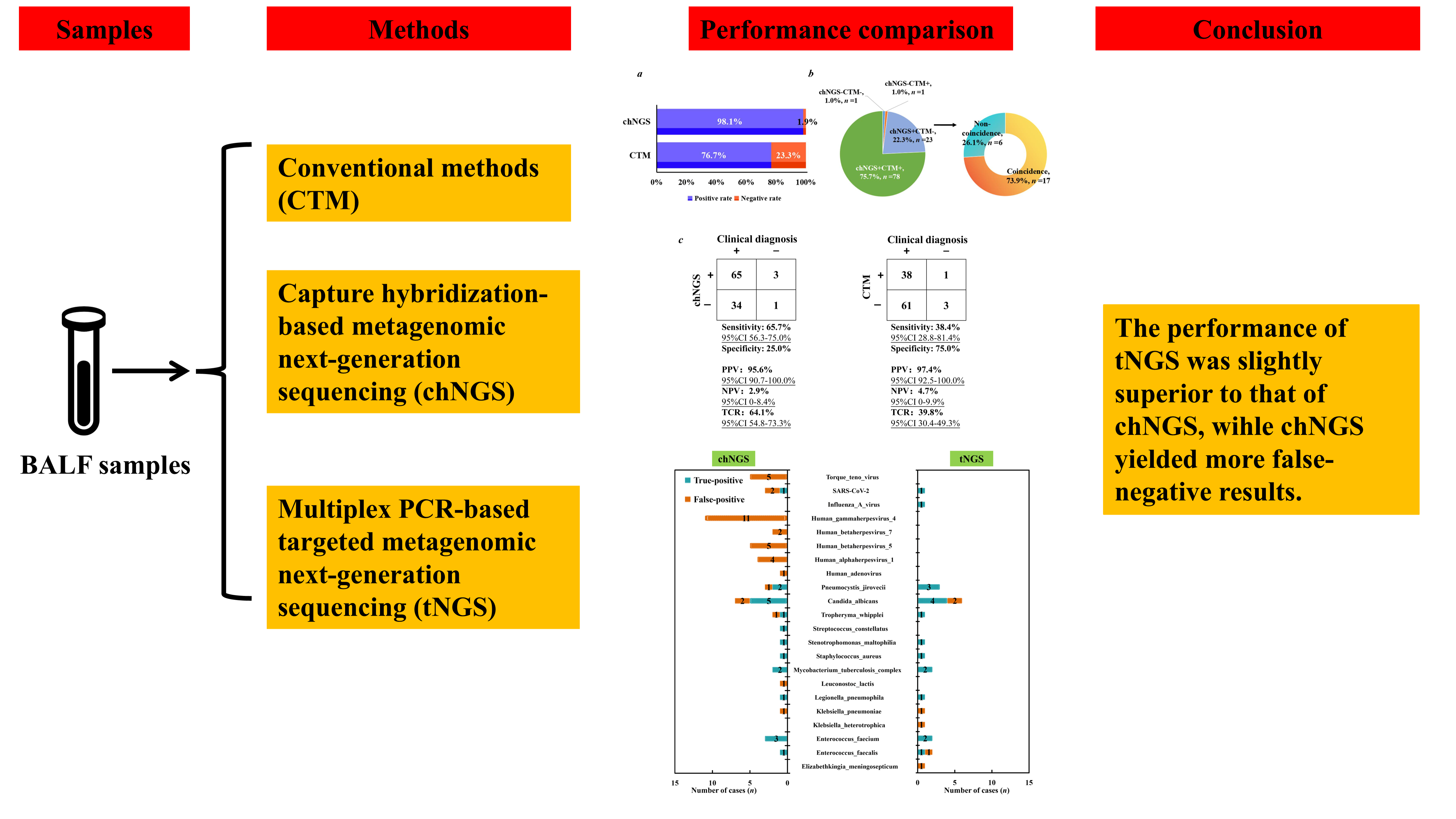

Supplement: Graphical abstract — Visual diagram of the study. [file spectrum.01713-24-s0002.tif]
